# Improved the Impact of SST for HY-2A Scatterometer Measurements by Using Neural Network Model

**DOI:** 10.3390/s23104825

**Published:** 2023-05-17

**Authors:** Jing Wang, Xuetong Xie, Ruru Deng, Jiayi Li, Yuming Tang, Yeheng Liang, Yu Guo

**Affiliations:** 1 School of Geography and Planning, Sun Yat-sen University, Guangzhou 510275, China; wangj725@mail2.sysu.edu.cn (J.W.); lijy286@mail2.sysu.edu.cn (J.L.); tangym7@mail2.sysu.edu.cn (Y.T.); liangyeheng@163.com (Y.L.); guoy87@mail2.sysu.edu.cn (Y.G.); 2 School of Geography and Remote Sensing, Guangzhou University, Guangzhou 510006, China; xiexuetong@gzhu.edu.cn; 3 Guangdong Engineering Research Center of Water Environment Remote Sensing Monitoring, Guangzhou 510275, China; 4 Guangdong Provincial Key Laboratory of Urbanization and Geo-Simulation, School of Geography and Planning, Guangzhou 510275, China; 5 Southern Marine Science and Engineering Guangdong Laboratory (Zhuhai), Zhuhai 528406, China

**Keywords:** ocean remote sensing, scatterometer, sea surface temperature, backscatter coefficient

## Abstract

The variation of sea surface temperature (SST) can change the backscatter coefficient measured by a scatterometer, resulting in a decrease in the accuracy of the sea surface wind measurement. This study proposed a new approach to correct the effect of SST on the backscatter coefficient. The method focuses on the Ku-band scatterometer HY-2A SCAT, which is more sensitive to SST than C-band scatterometers, can improve the wind measurement accuracy of the scatterometer without relying on reconstructed geophysical model function (GMF), and is more suitable for operational scatterometers. Through comparisons to WindSat wind data, we found that the Ku-band scatterometer HY-2A SCAT wind speeds are systemically lower under low SST and higher under high SST conditions. We trained a neural network model called the temperature neural network (TNNW) using HY-2A data and WindSat data. TNNW-corrected backscatter coefficients retrieved wind speed with a small systematic deviation from WindSat wind speed. In addition, we also carried out a validation of HY-2A wind and TNNW wind using European Center for Medium-Range Weather Forecasts (ECMWF) reanalysis data as a reference, and the results showed that the retrieved TNNW-corrected backscatter coefficient wind speed is more consistent with ECMWF wind speed, indicating that the method is effective in correcting SST impact on HY-2A scatterometer measurements.

## 1. Introduction

Satellite-borne scatterometers are active microwave sensors that provide real-time observations of the global sea surface wind field in all weather conditions. However, scatterometer observations can be affected by non-wind factors leading to a decrease in wind measurement accuracy, and SST is one of the factors that cannot be neglected. Therefore, how to clarify the impact of SST on scatterometer wind measurements and how to remove the impact of SST are important research directions for improving the scatterometer wind measurement capability. This study analyzed and summarized the characteristics of the effect of SST variation on backscatter coefficients measured by HY-2A scatterometer, and established a neural network model to remove the effect of SST from the backscatter coefficients. Unlike the methods that require large amounts of data and long-term observations to reconstruct the GMF, this neural-network-based technique provides a new insight for improving the accuracy of scatterometer wind measurements.

In 1978, NASA launched the SeaSAT-A satellite, which carried the first satellite-borne scatterometer, the SASS (SeaSat Scatterometer) [1]. Over the past 40 years, various countries and institutions have launched satellite-borne scatterometers at different frequencies (C-band or Ku-band) and researchers have conducted various studies and practices on satellite-borne scatterometers, leading to continuous improvements in scatterometer wind measurement technology. Sea surface wind fields measured using scatterometers are now applied in a variety of ocean and climate observations and are an important source of information for studying processes in the ocean environment, such as waves and ocean circulation [2]. Sea surface wind fields measured using satellite-borne scatterometers have also been assimilated into numerical weather prediction (NWP) to provide data support to respond effectively to marine hazards such as typhoons and storm surges [3,4].

The scatterometer sensor transmits a microwave signal to the ground and the received echo is typically characterized as the normalized radar cross section (NRCS), also known as the backscatter coefficient or σ^0^. The surface wind causes ripples on the sea surface, and the echo from the sea surface depends on these small centimeter waves (or gravity-capillary waves), which is fundamental for the relationship between the backscatter coefficient and the sea surface wind [5,6]. The GMF describes the relationship between the backscatter coefficient and the sea surface wind [7] and is an essential basis of the inversion of the wind field by the backscatter coefficient. It is difficult to construct an accurate physical GMF model because the relationship between the sea surface wind vector and sea surface geometry is not fully defined and the mechanism of the interaction between electromagnetic waves and the sea surface is very complex. Some researchers have proposed physical models based on wave parameters [8,9,10]; however, the GMFs currently used by operational satellite-borne scatterometers are empirical models [11] based on numerical statistics. Continuous research and improvement of GMFs has led to frequent updates to GMFs; for example, QSCAT-1, NSCAT-2, and NSCAT-4 in the Ku-band, CMOD4, CMOD5, and CMOD7 in the C-band, etc. [12,13,14,15]. The GMF usually only considers factors directly related to the backscatter coefficient such as sea surface wind speed, wind direction, polarization, and angle of incidence, while neglecting secondary factors such as waves, rain, sea surface salinity (SSS), and sea surface temperature (SST) [16].

The backscatter coefficient represents the roughness of the sea surface at the scale of gravity capillary waves, which are not only dominated by wind but also modulated by SST [17]. SST directly influences sea–air exchange and ocean viscous dissipation, which in turn affect wave generation and dissipation [18]. Liu confirmed the non-negligible effect of SST on wind speed by comparing SASS and ship-reported wind speeds [19]. The effect of SST on sea surface winds is usually related to temperature gradients and spatial scales [20]. SST is usually negatively correlated with sea surface wind speed variability at a large spatial scale [21,22,23]. Small et al. showed that SST perturbations are typically positively correlated with sea surface wind speed at small-to-medium scales of 100 to 1000 km [24]. O’Neill et al. studied the response of sea surface winds to SST measured using scatterometers at mid-latitudes and showed that when the SST changes, not only is the wind speed affected, but the wind direction also changes [25]. The results of quality tests on scatterometers of different bands show that sea surface temperature is the main reason for the difference in wind speed between C- and Ku-band scatterometers [26]. Bentamy et al. confirmed that C-band scatterometers are less sensitive to the effects of SST, while Ku-band scatterometers are more prominently affected by SST [18]. The backscatter coefficient response to SST varies for different polarizations, and Wang et al. showed that the V-polarization backscatter coefficient is more sensitive to SST effects [26]. Nghiem and Li analyzed the SST effects on the H- and V-polarization data using a high-resolution airborne Ku-band scatterometer for the Gulf Stream frontal temperature study and showed that the difference between the V-polarization backscatter coefficients can be larger than 5 dB at a 9 °C temperature difference, while the difference between the H-polarization backscatter coefficients is smaller [27].

Ku-band scatterometers have the characteristic of being susceptible to the influence of secondary factors such as SST; therefore, researchers have tried to improve Ku scatterometer wind fields inversion accuracy by improving the GMF. These methods usually use wind field data that are not sensitive to sea surface temperature as a reference for constructing the GMF; for example, the C-band scatterometer is less affected by sea surface temperature, so Wang et al. used the wind field data from the C-band scatterometer ASCAT to build a GMF including SST for the RapidScat scatterometer; the wind speed deviation obtained by using this model is less dependent on SST and the wind speed probability density function (PDF) is closer to the ASCAT wind speed [28]. Xie et al. chose the ECMWF reanalysis data as a reference and proposed an SST-dependent GMF for the Ku-band scatterometer HY-2A SCAT to improve its wind measurement accuracy [29]. The above studies are based on improving GMFs to improve the accuracy of scatterometer inversion of sea surface wind fields, and correcting for the backscatter coefficients themselves is also a feasible method. Academics have also attempted to separate the effects of SST on the backscatter coefficient using the theoretical analysis method, and there have been some achievements. The use of radar imaging models to evaluate and analyze the effects of SST on C- and Ku-band scatterometers is the theoretical basis provided by Grodsky et al. for improving the effects of SST [30]. Additionally, the analytic model developed using the combined wave spectrum and second-order small slope approximation model by Du et al. provides insights into the SST effects on ocean scattering and wind retrieval at various frequencies [31].

The effect of nonwind factors on the backscatter coefficient is usually nonlinear, and the complex backscatter mechanism increases the difficulty of separating these factors. Machine learning has the ability to process complex processes for specific models and can be applied to scatterometer inversion of sea surface wind fields. However, the results of machine learning are strongly dependent on the input data, and the machine learning training process requires a large amount of stable and well-distributed data, which are related to the final performance of the machine learning model. In addition, the machine learning process is opaque, so only the predicted results can be provided, but a clear picture of the prediction process cannot be provided. Machine learning has been widely used in sea surface wind field inversion, and Xu et al. used a support vector machine to map the sea surface wind field and also investigated rain rate scatterometer inversion [32]. Deep learning is a type of machine learning that uses deep neural networks to process the model in a more complex manner and thus deepen the model’s understanding of the data. Neural network models have also been applied to scatterometer inversion of wind fields. Stiles and Dunbar proposed a neural network-based method to improve the effect of rain on scatterometer wind measurements, which can map the sea surface wind field directly from the backscatter coefficient without using the GMF [33]. Xie et al. used a neural network to correct the backscatter coefficients affected by rain to improve the accuracy of the retrieved sea surface wind speed under rainy conditions [34]. The above machine learning-related scatterometer wind vector inversion studies inspired us to apply a neural network to the technique of correcting the effect of SST on the backscatter coefficient, which is mainly for the HY-2A scatterometer in the Ku-band.

The article is organized as follows. Section 2 describes the HY-2A level 2, WindSat, and ECMWF data used for the experiments, the data matching strategies, and the data preprocessing. The methods section describes the neural network technique and the wind field inversion method used in this study. In Section 3, we present the backscatter coefficient analysis results using WindSat data as a reference, and we double-validate the SST correction results of TNNW using WindSat data and ECMWF data. In Section 4, we explain the advantages and limitations of neural networks in this study and explored the mechanism of SST effects on scatterometer wind measurements. In Section 5, we present the conclusions of this study.

## 2. Datasets and Methods

### 2.1. Datasets

#### 2.1.1. HY-2A Data

The sun-synchronous orbit satellite HY-2A is a Chinese oceanographic satellite, also known as “China Ocean Satellite 1” (COS-1), that was launched on 16 August 2011. The satellite was developed by the China Academy of Space Technology (CAST) and is operated by the National Satellite Ocean Application Service (NSOAS) of China. The HY-2A is equipped with the Ku-band scatterometer HY-2A SCAT. HY-2A SCAT is a conically scanning pencil-beam scatterometer with an HH-polarized inner beam at an angle of incidence of 41° and a VV-polarized outer beam at an angle of incidence of 48°. HY-2A SCAT’s one-track scan data are divided into 1624-row and 76-column grids, each with a resolution of 25 × 25 km. These grids are called wind vector cells (WVCs) and the data contained in them are all the scatterometer measurements in the coverage of that grid. Each HY-2A L2A WVC records information including measured backscatter coefficients and associated parameters such as polarization, angle of incidence, angle of azimuth, observation time, and geographic location. In this study, we used HY-2A L2A products, provided by NSOAS from 1 January 2013 to 30 December 2013, as experimental data.

#### 2.1.2. WindSat Data

WindSat is a polarimetric microwave radiometer aboard the Department of Defense Coriolis satellite launched in June 2003 that provides the observation of sea surface parameters by measuring the radiometric brightness temperature of the sea surface. The WindSat radiometer operates in five discrete bands: 6.8, 10.7, 18.7, 23.8, and 37.0 GHz. All are fully polarimetric except the 6.8 and 23.8 GHz channels, which are only dual-polarized (vertical and horizontal) [35]. The major advantage of WindSat over previous satellite-borne radiometers such as SSM/I is its ability to measure both sea surface wind speed and direction with a multi-polarimetric instrument. The WindSat wind vector and SST data used in this study are data products provided by Remote Sensing System (RRS). RRS’s WindSat data products include parameters such as sea surface temperature, a variety of surface wind speeds and directions, atmospheric water vapor, cloud liquid water, and rain rates. By comparing the WindSat wind vectors with anemometer winds from oil platforms, Manaster et al. demonstrated that WindSat wind vectors are very consistent with the in situ data [36], indicating that the accuracy of the WindSat wind vector is reliable. The WindSat wind vector provided by RRS is a 10 m high surface wind, which is consistent with the HY-2A-retrieved sea surface wind. RRS divides the global surface into a grid of 1440 rows and 720 columns at a resolution of 0.25°, providing both near-real-time and “final” data, and we used the final data in this study.

#### 2.1.3. ECMWF Data

ECMWF regularly uses its forecast models and data assimilation systems to “reanalyze” archived observations to create global datasets describing the recent history of the atmosphere, land, and oceans. The ECMWF is now recognized as a world leader in numerical prediction results, due in large part to its advanced data assimilation system. The reanalysis of data uses assimilation methods to combine model data with observations from around the world to form a globally complete and consistent data set. In this study, the latest ECMWF reanalysis data, ERA5 [37], were used. ERA5 is a reanalysis dataset generated using 4D Var data assimilation and model forecasts in the ECMWF integrated forecast system (IFS) CY41R2. The ERA5 dataset includes a wide range of atmospheric and oceanic parameters, which can be selected to suit the temporal and spatial resolution as needed. We used SST and 10-m-high wind speed u- and v-component data with a 0.25° spatial resolution and a 1 h temporal resolution in our study. In addition, ECMWF produces global ocean and sea ice reanalysis data (ORAS5: Ocean Reanalysis System 5) [38] using 3D-Var FGAT (first guess at appropriate time) assimilation technology, which provides monthly average data on ocean parameters including sea surface temperature, sea surface salinity, sea ice concentration, and sea-level anomalies. Although ORAS5 is specifically designed to assimilate satellite observations of the ocean, we finally chose ERA5 data because ORA5 provides monthly averaged data and cannot provide hourly data in the same way as ERA5 data.

#### 2.1.4. Collocations

In this study, we used a variety of data involved in modeling and validation, and there are spatial and temporal differences between the different data; therefore, a data matching process was required. The spatiotemporal resolution of different data is not consistent, so the data matching strategy has to be conducted based on the characteristics of the data. First, we needed to match the WindSat data with the HY-2A data, which were used for neural network modeling and validation. The WindSat data are real-time data and report sea surface wind and sea surface temperature. WindSat is a polar-orbiting satellite radiometer; the measured sea surface data are distributed in swathes and the recording time of each swath varies greatly. There is an interval between WindSat data swathes, so the spatiotemporal interpolation of the data may affect authenticity. We used the nearest neighbor method to select the WindSat data whose geographic coordinates are less than 12.5 km away from the latitude and longitude of the HY-2A cell center for matching. The dynamically changing characteristics of sea surface wind require that the time difference between the matched data be as small as possible. Setting too strict a temporal window affects the amount of matched data, so we set the matching temporal window for HY-2A and WindSat to 10 min to reduce the time difference effect while ensuring the amount of data used for experiments. In addition, to reduce the impact of rain, we excluded data with a WindSat rain rate greater than 0 mm/hr. Following the above data matching strategy, we obtained 5,532,254 pairs of HY-2A and WindSat matching data. Second, we matched the HY-2A and ECMWF data used for dual validation. In setting the temporal window, we used the same temporal window as when matching WindSat, i.e., excluding data with a time difference greater than 10 min between the two. Since ECMWF provides hourly global data, the u and v components of the sea surface wind at any latitude and longitude can be obtained by interpolation. In the matching process, we obtained the corresponding ECMWF wind and SST data by interpolation using the geographical coordinates of HY-2A wind WVCs. The HY-2A and ECMWF datasets do not include the portion of rain rate over 0 mm/hr. Following the above matching strategy, we finally obtained 12,703,682 pairs of HY-2A- and ECMWF-paired data.

#### 2.1.5. Preprocessing

The GMF describes the relationship between the sea surface radar backscatter coefficient, radar observation parameters, and ocean environmental parameters with the sea surface wind speed and direction, and the scatterometer wind retrieval is based on the GMF. One approach for deriving the scatterometer GMF is to empirically correlate the radar measurements with the numerical weather model wind fields, which was utilized to obtain the model function for the operational scatterometers [39]. The empirical GMF typically expresses the backscatter coefficient as a Fourier series:

(1) $$\sigma^{0}=A_{0}\left(U;\theta_{inc},p,\lambda \right)+A_{1}\left(U;\theta_{inc},p,\lambda \right)\cos\left(\phi \right)+A_{2}\left(U;\theta_{inc},p,\lambda \right)\cos\left(2\phi \right).$$

In Formula (1), *σ*^0^ is the backscatter coefficient, *U* is the wind speed, *ϕ* is the relative wind direction, *θ_inc_* is the incidence angle, *p* is the polarization, and *λ* is the wavelength of the radiation. The parameters *A*_0_, *A*_1,_ and *A*_2_ are generally functions of incidence angle, radar frequency, and polarization.

The neural network’s training requires the use of simulation backscatter coefficients. The process of calculating the simulation backscatter coefficient is to input the wind direction, wind speed, azimuth, and polarization information into the GMF to obtain the corresponding backscatter coefficient. Among them, the azimuth and polarization information are used from the HY-2A measurements, and the wind speed and direction are provided by the matched WindSat data. To be consistent with the model used in the HY-2A operational inversion, the GMF used throughout the experiment was the NSCAT-2 GMF. The NSCAT-2 GMF is the GMF developed for the NSCAT-2 scatterometer that ended operation in 1997, while subsequent Ku-band scatterometers such as QuikSCAT and ASCAT still follow this GMF model with some modifications based on the differences in radar instrumentation and measurement techniques. Additionally, some new Ku-band GMFs such as NSCAT-4 have also chosen NSCAT-2 as the basis for their upgrades. Although NSCAT-2 is not a very new GMF, it has been tested using different operational scatterometers for many years, and choosing it as the inversion model obtains a relatively stable result.

### 2.2. Methods

The main objective of this study was to improve the accuracy of HY-2A scatterometer wind measurements by removing the influence of SST on the backscatter coefficients. To make our objective more understandable, the effect of SST on scatterometer inversion is expressed using a temperature-dependent wind retrieval error formula [30]:

(2) $$dW=\frac{\sigma^{0}\left(W,T\right)-\sigma^{0}\left(W,T_{0}\right)}{{\frac{\partial \sigma^{0}}{\partial W}|}_{T_{0}}},$$

where *σ*^0^ (*W*, *T*) represents the backscatter coefficient measured by the scatterometer, *W* is the given wind speed that depends on SST, *T*. *σ*^0^ (*W*, *T*_0_) is the backscatter coefficient provided by the GMF, and, since the GMF does not take temperature into account, *T*_0_ is equal to the global mean SST = 19 °C. The difference between the measured backscatter coefficients and the GMF backscatter coefficients was reduced by the correction of the neural network in order to reduce the wind velocity error under different temperature conditions.

**Figure 1:**
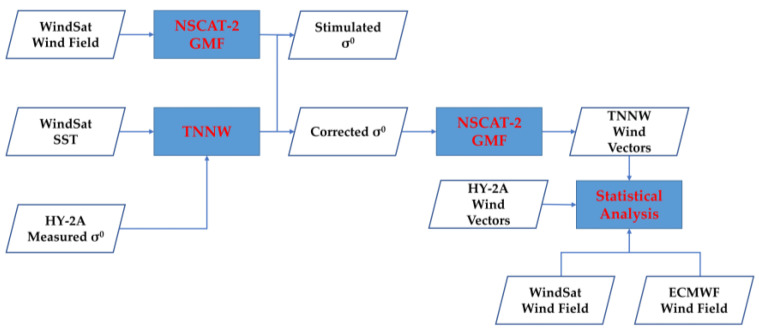
Flow chart of the HY-2A scatterometer neural network SST effect correction.

The main methods used in this study were neural network modeling and sea surface wind inversion. Figure 1 shows the research flow chart of the study. The main role of the neural network model we built is to improve the effect of SST on the backscatter coefficient and, for ease of understanding, we named this model the temperature neural network, which is abbreviated as TNNW herein. TNNW is a neural network model built using the BP algorithm. In training the TNNW, the backscatter coefficients measured using HY-2A and the SST measured using WindSat were used as inputs to the neural network and the simulation backscatter coefficients calculated from WindSat wind field data were used as outputs. In the surface wind retrieval stage, we used the NSCAT-2 GMF mentioned in Section 2.1.5 as a basis for inverting the backscatter coefficient to wind speed and wind direction using the MLE method. Finally, we used WindSat winds and ECMWF winds as references to evaluate the accuracy of the inversion results.

#### 2.2.1. Neural Network Modeling

In this study, we used a simple neural network approach, a back propagation (BP) neural network, for modeling the TNNW. The BP neural network was proposed by a group of scientists led by Rumelhart and McCelland in 1986 as a multilayer feedforward network trained according to the error backpropagation algorithm and it is one of the most widely used neural network models. The structure of a BP neural network is characterized by full multilayer connectivity with error back propagation.

The data distribution must be taken into account when training neural networks. If the data distribution is not balanced enough, the features that account for fewer modeling data are ignored during training, affecting the accuracy of the model. We classified the matched HY-2A and WindSat data pairs using polarization, wind speed, relative wind direction, and SST. First, the training data were divided into two parts according to polarization and were used to build neural network models with different polarizations. Then, the training data were classified according to the wind speed range 4–15 m/s, step 0.1 m/s; relative wind direction range 0–180°, step 2°; and SST range 0–30 °C, step 1 °C. Finally, after assigning each matched datum to the corresponding category, 10 of the classified data were randomly selected as training data under this category, and if the tallied numbers of data in this category were less than 10, the data in this category were filled up to 10 using randomly repeated sampling. The final HH polarization data pairs and VV polarization data pairs we obtained for training were 333,870 and 334,090 pairs, respectively.

**Figure 2:**
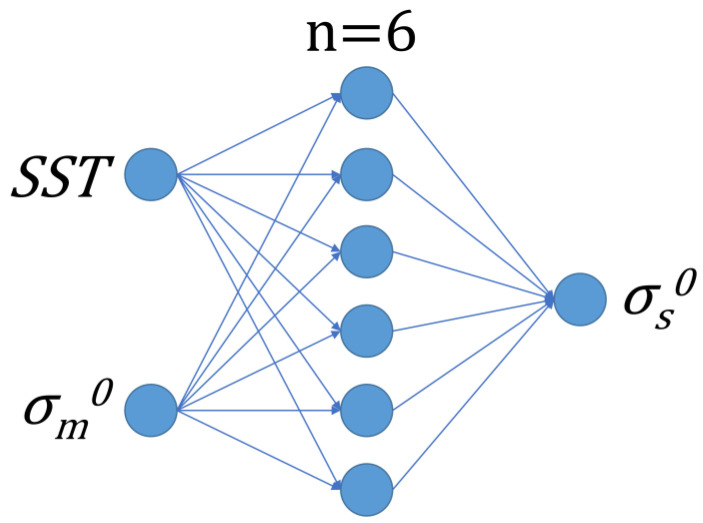
Topological structure diagram of BP neural network.

Figure 2 shows the topology of the BP neural network used in this study, which contains three feedforward layers, namely the input layer, the middle layer (also called the hidden layer), and the output layer. The input layer is the backscatter coefficient measured using HY-2A, represented as *σ*_m_^0^, and the sea surface temperature measured by WindSat, represented as SST. The hidden layer has six neurons and the output layer is the simulation backscatter coefficient that is calculated using WindSat wind data, represented as *σ*_s_^0^. The neurons in each layer are only fully connected to the neurons in adjacent layers, and there is no connection between neurons in the same layer and no feedback connection between neurons in each layer, forming a feed-forward neural network system with a layered structure. The BP neural network used in this study, with an input layer containing two nodes and an output layer containing one node, can be viewed as a mapping of a two-dimensional vector to a one-dimensional vector.

The BP algorithm stores the learning results in the connection weights of neurons, thus enabling intelligent learning. However, its operation requires a large amount of training data and continuous iterative learning, which implies a long learning time. The initial values of weights and biases may affect the ability of BP neural networks to generalize to new data. If the BP neural network has too many hidden layers or neurons, then it may lead to overfitting. The BP algorithm has the risk of becoming stuck in local optima, which are suboptimal solutions that are not the global minimum of the loss function. Combining the above analysis of BP neural networks, we constructed a simple model with one hidden layer, including five neurons. In the modeling process, we also tried to add more hidden layers and neurons; the test results show that the performance of the complex network structure is not better than the simple neural network structure, but also increases the possibility of training failure. In order to avoid the BP algorithm falling into local minima, we initialized the bias with 0, initialized the weights randomly, set the learning steps from 0.9 to 0.1, and set the maximum number of iterations to 500. Training ended only when the total network error was no longer decreasing or when the maximum number of iterations was reached. Using repeated training and many tests, we finally obtained the TNNW. Although the TNNW can be used to improve the SST effect in the backscatter coefficient, it is difficult to know how the TNNW predicts the effect of SST since the neural network is a “black box”.

#### 2.2.2. Wind Vector Retrieval Algorithm

For the retrieval of sea surface wind vectors, this study used a method common to operational scatterometers. First, the model values corresponding to several observations in the WVC of the scatterometer were retrieved based on the known GMF. Then, the inversion algorithm based on the maximum likelihood estimation (MLE) method was used to obtain the objective function of the model values, and the corresponding wind vector fuzzy solution was searched according to the objective function. Finally, the true wind vector was obtained using fuzzy solution removal.

The GMF model value retrieval process is described in Section 2.1.5, and here we focused on the retrieval method, which is the MLE based on Bayesian distribution development [40,41,42]. The MLE method is currently considered to be the best method for wind vector inversion, and its objective function is expressed as follows:

(3) $$J=-\sum_{i=1}^{N}\frac{{\left(\sigma_{0i}-\sigma_{m}\left(w,\phi_{i}\right)\right)}^{2}}{Var{\left(\sigma_{m}\right)}_{i}}+\ln\left(Var{\left(\sigma_{m}\right)}_{i}\right).$$

In Formula (3), *σ*_0*i*_ represents the measured value of the backscatter coefficient, *σ_m_*(*w*, *ϕ_i_*) represents the GMF’s model value, *N* represents the number of backscatter coefficient measurements, and *Var*(*σ_m_*)*_i_* represents the measurement variance, which depends on the antenna and the position of the WVC. Usually, numerical methods are used to solve the above objective function and the objective function values are calculated point-by-point in the whole wind speed–wind direction two-dimensional space according to a certain search interval and compared to find local extremum points. The wind speed and direction corresponding to the local extremum obtained using maximum likelihood estimation is called the potential wind velocity solution (known as “ambiguities”).

There are usually several ambiguities in a WVC, and one of them is closest to the real wind. The process of finding the “closest” solution to the WVC is called ambiguity removal (AR). Several ambiguity removal techniques have been suggested by experts. In the HY-2A standard inversion process, the circular median filter is used as an AR method. The circular median filter takes the reference wind field as the initial field; that is, the wind vector solution closest to the wind direction of the reference wind field is selected as the current real wind velocity solution from the ambiguities of each WVC. Then, the wind velocity closest to the wind velocity solutions in the surrounding WVCs is found as the true wind velocity using a circular median filter. After multiple iterations, the true wind velocity solution no longer changes and the final true wind field is obtained [43].

#### 2.2.3. SST Impact Assessment

Since the variation in the backscatter coefficient with SST is related to the wind speed, the SST-influenced backscatter coefficient variation Δ*σ* is defined to clarify the response characteristics of the backscatter coefficient to the variation of SST and wind speed. Δ*σ* represents the difference in backscatter coefficient between SST of 30 °C and 0 °C at different wind speeds:

(4) $$\Delta \sigma \left(v\right)=\sigma_{m}\left(v,\;30\;℃\right)-\sigma_{m}\left(v,\;0\;℃\right).$$

In Formula (4), *v* is the wind speed and *σ_m_* is the mean value of the backscatter coefficient measured using HY-2A.

Δ*σ* reflects the amount of variation in the effect of SST on the backscatter coefficient, but the same amount of variation has significantly different effects at different wind speeds. This is because the variation in the backscatter coefficient corresponding to the wind speed decreases as the wind speed increases. This means that the same magnitude of change in the backscatter coefficient has much less effect at low wind speeds than that at high wind speeds. Therefore, to better investigate the effect of SST on wind speed, we defined the temperature sensitivity *s* as a function of:

(5) $$s\left(v\right)=\frac{\sigma_{m}\left(v,\;30\;℃\right)-\sigma_{m}\left(v,\;0\;℃\right)}{d\left(v\right)}.$$

In Formula (5), *d* is the difference between the mean values of the backscatter coefficients of the upper and lower 1 m/s wind speeds. The higher the temperature sensitivity value, the larger the SST effect’s magnitude. Therefore, the temperature sensitivity can better describe how much the SST-influenced backscatter coefficient contributes to the wind speed.

## 3. Results

### 3.1. Comparative Analysis of Backscatter Coefficient

**Figure 3:**
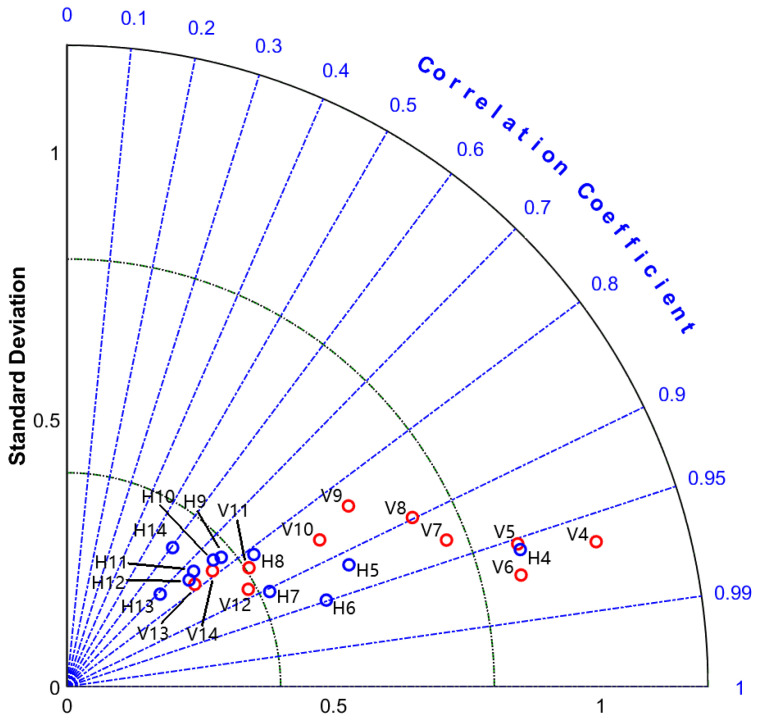
Taylor diagram of HY-2A measured backscatter coefficient. The red circles represent VV polarization and the blue circles represent HH polarization.

Before analyzing the specific effect of SST on the HY-2A backscatter coefficient, we wish to illustrate the relationship between the variation of the HY-2A backscatter coefficient and the SST using some statistical indicators. We calculated the standard deviation of the HY-2A backscatter coefficient and the correlation coefficient between the backscatter coefficient and the sea surface temperature for different polarizations, respectively. The HY-2A data of different polarizations with WindSat wind speed of 4–14 m/s and SST range of 0–30° were divided into 22 groups according to a wind speed interval of 1 m/s. The standard deviation of the backscatter coefficients in each group was calculated first, and then the mean values of the backscatter coefficients in each group were calculated separately in 2 °C steps. Finally, the correlation coefficients between these averaged backscatter coefficients and SST were calculated. Figure 3 shows the Taylor diagram of the calculation results. The red circles represent the VV polarization, the blue circles represent the HH polarization, the label “V4” indicates the VV polarization datapoint with a wind speed of 4 m/s, and “H4” indicates the HH polarization datapoint with a wind speed of 4 m/s with other labels and so on. It can be seen from this figure that the correlation coefficients between the mean values of HY-2A backscatter coefficients and sea surface temperature for both HH and VV polarizations are greater than 0.6, and some data are greater than 0.95, indicating that the variation in HY-2A backscatter coefficients for either polarization is positively correlated with SST. Comparing the positions of red circles and blue circles, we can find that the correlation coefficients of the backscatter coefficients of VV polarization and temperature are mostly larger than 0.8, while the correlation coefficients of blue circles are relatively smaller, indicating that the VV polarization data are more likely to change with sea surface temperature. Additionally, the correlation coefficient decreases as wind speed increases, indicating that the SST’s effect on the backscatter coefficient may be less than that of a lower wind speed when the wind speed is higher, but this does not mean that the SST’s effect on the inverse wind speed shows the same trend. The red circle in the figure has a larger standard deviation than the blue circle, which indicates that the VV polarization data are more discrete, and the data dispersion decreases with the increase in wind speed regardless of polarization, but this does not mean that the variation in wind speed decreases with the increase in wind speed, which is because the same amount of backscatter coefficient variation has different effects on different wind speeds. We infer that the reason for the variation in the standard deviation of backscatter coefficient is related to the instrument noise of scatterometer and the atmospheric influence, i.e., the instrument calibration and noise account for a larger part of the backscatter coefficient observed by HY-2A when the wind speed is low, thus leading to an increase in the random error.

**Figure 4:**
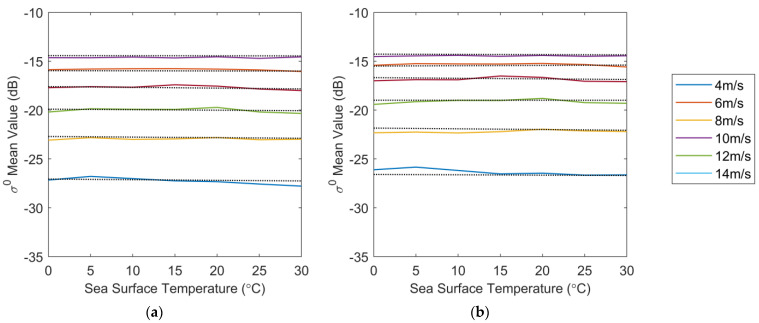
Mean value of backscatter coefficient at different wind speeds as functions of SST. (**a**) for HH pol, (**b**) for VV-pol. Solid lines represent TNNW-corrected *σ*^0^ and dotted lines represent the WindSat backscatter coefficient *σ*^0^.

To compare the relationship between the TNNW corrected backscatter coefficients and the WindSat simulation backscatter coefficients, we classified the backscatter coefficients according to fixed wind speed and fixed SST intervals and calculated the mean value of the backscatter coefficients in each category to compare the differences between the TNNW-corrected backscatter coefficients and the WindSat simulation backscatter coefficients. The mean backscatter coefficient at WindSat wind speeds of 4–14 m/s as a function of SST for HH polarization and VV polarization are shown in Figure 4a,b, respectively. The solid line in Figure 4 is the TNNW-corrected backscatter coefficient, the dotted line is the WindSat simulation backscatter coefficient, (a) is the HH polarization, and (b) is the VV polarization. The solid and dotted lines of the different polarizations in Figure 4 are slightly different but no large deviations were observed. The solid lines in the figure basically maintain the horizontal state, without obvious deviation and tilt with the change in SST, and the two have good agreement. This indicates that the TNNW can better extract the change rule of the backscatter coefficient with SST and can correct the influence of SST on the backscatter coefficient. The corrected backscatter coefficient is closer to the backscatter coefficient simulated using WindSat wind.

In order to quantify the agreement between the TNNW-corrected backscatter coefficients and WindSat-simulated backscatter coefficients, we calculated their correlation coefficients, and Table 1 shows the results of the grouping calculations according to the WindSat wind speed range of 4–14 m/s in 1 m/s steps. In general, most of the TNNW backscatter coefficients are in good agreement with the WindSat-simulated backscatter coefficients, and the correlation coefficients between them are basically greater than 0.8. For the analysis of specific wind speed segments, we found that the correlation coefficients between the TNNW backscatter coefficients and the WindSat-simulated backscatter coefficients are lower under low wind speed conditions, and the VV polarization is lower than the HH polarization. The reason for this situation is estimated to be that the instrument noise and atmospheric influence are too strong under low wind speed conditions, resulting in large random errors in this part of the data and, therefore, the TNNW cannot be fitted well.

**Figure 5:**
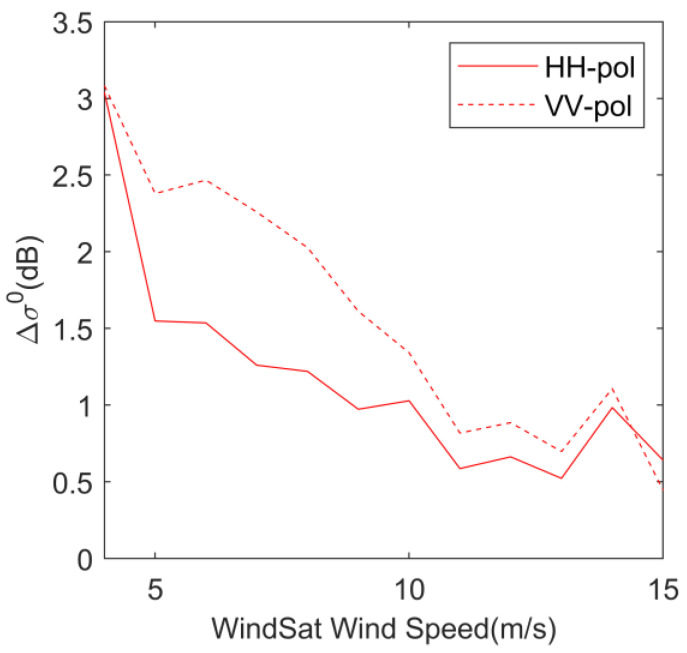
Δ*σ* as functions of WindSat wind speed. Solid lines represented HH-pol and dotted lines represented VV-pol.

We calculated the variation of HY-2A measured backscatter coefficient with SST following the method in Section 2.2.3. Figure 5 shows Δ*σ* as a function of WindSat wind speed, the solid line is HH polarization, and the dashed line is VV polarization. In Figure 5, when the wind speed is 4 m/s, the backscatter coefficient variation is the largest, and the Δ*σ* of HH polarization and VV polarization is more than 3 dB. With the increase in wind speed, the Δ*σ* starts to become smaller, and when the wind speed is 15 m/s, the Δ*σ* of HH polarization is 0.65 dB and the Δ*σ* of VV polarization is 0.49 dB. When the wind speed is low, the roughness of the sea surface is lower, and the backscatter coefficient is also lower. Therefore, compared with the high wind speed conditions where the roughness of the sea surface is large, the variation in the backscatter coefficient caused by SST accounts for a larger proportion of the backscatter coefficient observed using HY-2A, a situation occurs where the amount of backscatter coefficient variation is relatively larger at low wind speeds, and when the wind speed increases, the proportion of the effect caused by SST in the backscattering signal received by the scatterometer is relatively reduced. In addition, the Δ*σ* curve of VV polarization is on top of the Δ*σ* curve of HH polarization for most wind speed conditions, suggesting that SST may cause a larger variation in the VV polarization backscatter coefficient.

**Figure 6:**
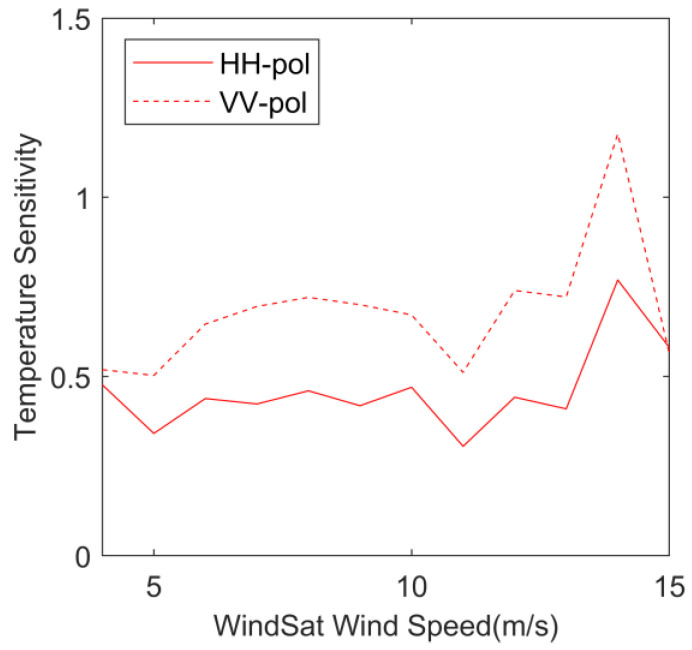
Temperature sensitivities as functions of WindSat wind speed. Solid lines represent HH-pol and dotted lines represent VV-pol.

Figure 6 shows the variation curve of temperature sensitivity with wind speed. The temperature sensitivity was calculated according to the method mentioned in Section 2.2.3. When the wind speed is 4 m/s, the temperature sensitivity of VV polarization and HH polarization is approximately equal to 0.5, which means that within the SST variation range of 0–30 °C, when the wind speed is 4 m/s, the change in temperature on the backscatter coefficient can cause a wind speed deviation of 0.5 m/s. When the wind speed is greater than 5 m/s, the VV polarization’s temperature sensitivity begins to increase, while the HH polarization temperature sensitivity curve fluctuates horizontally. When the wind speed is greater than 10 m/s, the temperature sensitivity of HH polarization and VV polarization decreases slightly, but when the wind speed reaches 12 m/s, the temperature sensitivity starts to increase again. When the wind speed reaches 14 m/s, the HH polarization and VV polarization temperature sensitivities reach their peak at 0.76 and 1.17, respectively. When the wind speed is 15 m/s, the temperature sensitivity of VV polarization and HH polarization decreases, which may be due to the fact that, when the SST is high, there are fewer data for high wind speeds, which affects the temperature sensitivity calculation results. Overall, the impact of SST on wind speed increases with the increase in wind speed; that is, the higher the wind speed, the more susceptible it is to the influence of SST, and the VV polarization temperature sensitivity is greater than that of the HH polarization, indicating that the VV polarization backscatter coefficient has a greater response to the change in SST.

### 3.2. Analysis of Retrieved Wind Velocities

The effect of the SST on the backscatter coefficient is eventually reflected in the sea surface wind inversion. In this subsection, we compare and analyze the results of HY-2A wind and TNNW-corrected wind using the WindSat and ECMWF wind fields, respectively.

#### 3.2.1. Comparison with WindSat Wind

**Figure 7:**
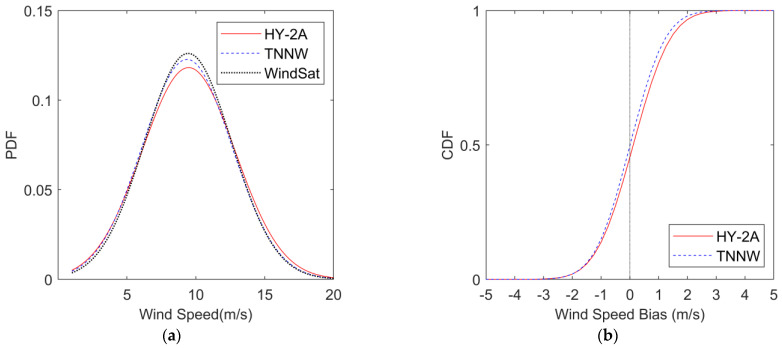
PDF plots and CDF Plots. (**a**) PDF as function of wind speed. The red solid line represents HY-2A, the blue dashed line represents TNNW and the black dotted line represents WindSat; (**b**) CDF as function of wind Speed bias with WindSat. The red solid line represents HY-2A and the blue dashed line represents TNNW.

We used half of the matched HY-2A and WindSat data to train the neural network, and the other half to analyze the effect of SST on the wind speed measured using the scatterometer and for examining the difference between the wind field retrieved from the TNNW-corrected backscatter coefficients and the WindSat wind field. Figure 7a shows the probability density function (PDF) of the HY-2A-retrieved wind speed, the TNNW wind speed, and the WindSat wind speed, from which it can be seen that the shapes of the TNNW wind speed and the WindSat wind speed are closer; the shape of the HY-2A wind speed, on the other hand, is significantly different from both, suggesting that the difference between HY-2A wind speed and WindSat wind speed is larger than that of the TNNW wind speed. Figure 7b shows the cumulative distribution functions (CDF) of the differences between HY-2A wind speed and TNNW wind speed with WindSat wind speed (HY-2A or TNNW wind speed minus WindSat wind speed). The red solid line represents the HY-2A bias, while the blue dashed line represents the TNNW bias; the difference between them is small and the blue dashed line has a smaller slope than the red solid line. The positions of the 0-value line intersecting the CDF curves of both HY-2A and TNNW are around 50%, meaning that both the HY-2A bias and the TNNW bias fall into positive and negative values with equal probability. Combining the PDF plots and the CDF plots, it can be seen that there is an obvious difference between the HY-2A wind speed and the WindSat wind speed; specifically, the HY-2A wind speed value is slightly larger than the WindSat wind speed. The TNNW-improved backscatter coefficient-retrieved wind speed is closer to the WindSat wind speed, without significant systematic deviation.

**Figure 8:**
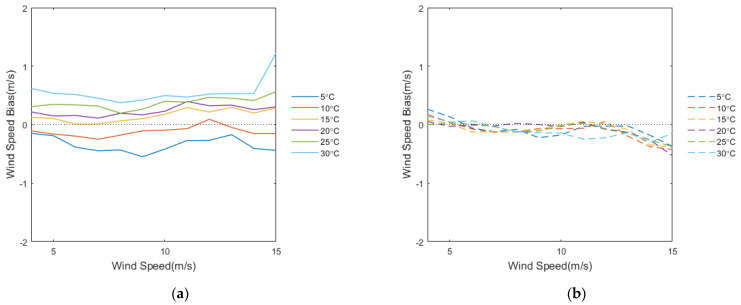
Wind speed bias of HY-2A and NNW as functions of WindSat wind speed under different SSTs. (**a**) represents HY-2A and (**b**) represents TNNW.

To further analyze the role of SST in the wind speed difference between WindSat and HY-2A, and to clarify the effect of the TNNW in improving this difference, we classified and compared the bias of HY-2A and TNNW with WindSat from 5 °C to 30 °C with a 5 °C interval according to the WindSat wind speed range of 4–15 m/s. Figure 8a,b show the bias of the HY-2A and the TNNW wind speeds with WindSat speed (HY-2A or TNNW wind speed minus WindSat wind speed) as a function of the WindSat wind speed under different SST conditions. From the figure, it can be seen that the deviation curve of the HY-2A-measured backscatter coefficient inversion wind speed under the low SST condition is below the 0-value line, indicating that the HY-2A wind speed is lower than the WindSat wind speed. As the SST increases, the mean deviation of HY-2A in the moderate and high wind speed segments starts to increase gradually. When the wind speed is equal to 12 m/s and the SST is equal to 10 °C, the HY-2A deviation curve crosses the 0-value line, indicating that the sea surface wind speed of the HY-2A is greater than the WindSat wind speed. With a further increase in temperature, the HY-2A wind speed bias reaches a maximum value of nearly 1.2 m/s when the SST reaches 30° and the WindSat wind speed is 15 m/s. The mean TNNW wind speed deviation curve, represented by the dashed line, fluctuates around the 0-value line for various wind speed and SST conditions, indicating that the impact of SST on the retrieved wind speed is reduced by correcting the backscatter coefficient using the TNNW. In addition, the value of the dashed line is slightly lower when the wind speed is high. It is speculated that the data used to train the TNNW are not sufficient under high wind speed conditions, so the wind speed bias is larger in this part but the maximum bias does not exceed 0.4 m/s.

**Figure 9:**
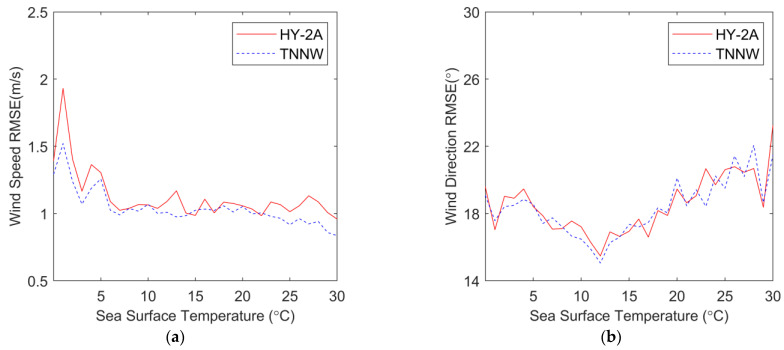
RMSEs of wind speed and wind direction as functions of the WindSat SST. (**a**) is the wind speed RMSE, (**b**) is the wind direction RMSE. Red solid lines represent HY-2A wind and blue dashed line represent TNNW wind.

To analyze the deviations between TNNW wind and HY-2A wind with WindSat wind, we calculated the RMSE of the wind direction and wind speed, and Figure 9 shows the RMSE of HY-2A and NNW wind speed and wind direction as functions of SST. Figure 9a shows the wind speed RMSE curve, where the blue dashed line represents TNNW wind, which is below the red solid line representing HY-2A wind, indicating that the difference between TNNW wind speed and WindSat wind speed is smaller. The wind direction RMSE curves in Figure 9b show that the HY-2A and TNNW wind direction deviation curves are very close to each other, indicating that the TNNW correction has little effect on wind direction and does not cause a new wind direction deviation. The maximum value of wind direction deviations appears at 30 °C, followed by 0 °C, indicating that both low and high temperature conditions are more likely to cause an increase in wind direction deviation.

#### 3.2.2. Validation Using ECMWF Data

**Figure 10:**
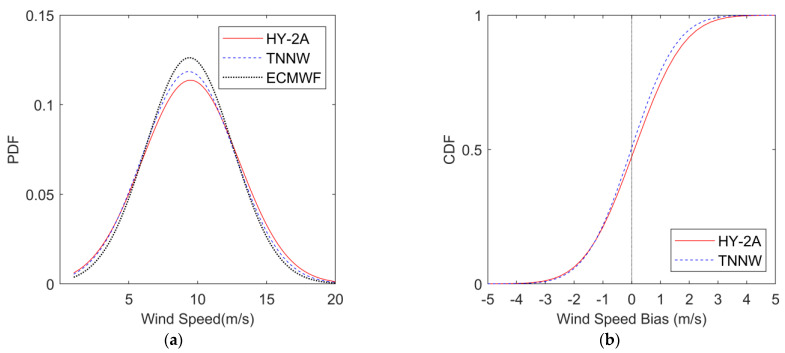
PDF plots and CDF Plots. (**a**) PDF as function of wind Speed. The red solid line represents HY-2A, the blue dashed line represents TNNW and the black dotted line represents ECMWF; (**b**) CDF as function of wind speed bias with ECMWF. The red solid line represents HY-2A and the blue dashed line represents TNNW.

To explore whether the difference in the comparison of the HY-2A inverse wind field and the WindSat wind field also appear in the comparison with other wind fields, and to analyze how much these differences are related to SST, we used ECMWF wind and SST data as a reference and analyzed the differences between HY-2A and ECMWF winds using the same method as for the WindSat data for comparison. Figure 10a shows the PDF curves of HY-2A wind speed, TNNW wind speed, and ECMWF wind speed. From the shape of the curves in the figure, the difference between the PDF shape of HY-2A and ECMWF is obvious, while the difference between TNNW wind and ECMWF wind is smaller. It shows that, although there is some deviation between TNNW wind speed and ECMWF wind speed, the deviation between HY-2A wind speed and ECMWF wind speed is larger. This is because TNNW is trained with WindSat wind and there is a slight difference between ECMWF and WindSat, so the output backscatter coefficient-retrieved wind speed is more biased towards WindSat wind. The distribution of CDF curves in Figure 10b is similar to that in Figure 7b, where the 0-value line almost equally divides the red solid line and the blue dashed line representing the CDF between HY-2A and TNNW wind speeds with ECMWF wind speed, indicating that the deviation distribution between TNNW and HY-2A wind speeds and ECMWF wind speed is more balanced and the systematic bias can be neglected. Comparing with Figure 7, it is clear that the differences between HY-2A and ECMWF wind speeds are similar to the differences between HY-2A and WindSat, while the wind speed consistencies between TNNW with ECMWF and WindSat are both better than those of HY-2A. However, the wind speed deviations between ECMWF wind with HY-2A wind and TNNW wind are larger than those with WindSat wind.

**Figure 11:**
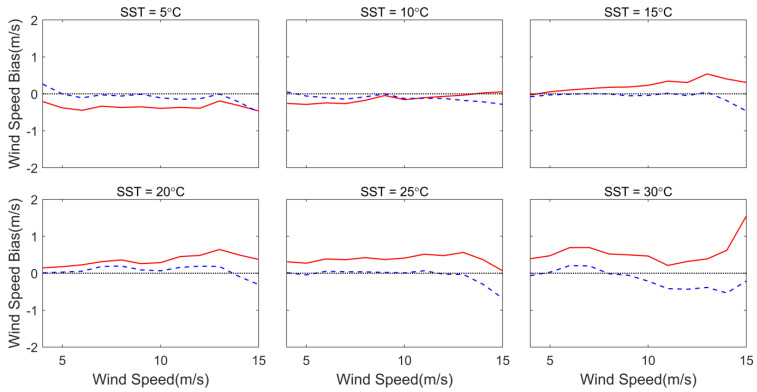
Wind speed bias of HY-2A and TNNW as functions of ECMWF wind speed under different SST. The red solid line represented HY-2A and the blue dashed line represented TNNW.

The wind speed difference between HY-2A and ECMWF shows the same pattern as the wind speed difference between HY-2A and WindSat. To observe whether this pattern is correlated with SST, we used ECMWF data for verification and evaluated the difference between the TNNW-improved backscatter coefficient inversion wind speed and ECMWF wind speed. The same wind speed and SST intervals as in Figure 8 were followed for Figure 11, except that the reference data were replaced with ECMWF data. The red solid line in Figure 11 is the mean wind speed bias curve of HY-2A and the blue dashed line is the mean wind speed bias curve of TNNW. The wind speed bias of HY-2A under low SST conditions is negative, which is consistent with the WindSat comparison. As the SST increases, the wind speed bias changes from negative to positive and gradually increases in the moderate and high wind speed segments and this change is also evident in Figure 8. The HY-2A wind speed bias in the low wind speed segments also increases with increasing SST but more slowly than in the moderate and high wind speed segments. Therefore, we speculate that the effect of SST on HY-2A wind speed underestimates the wind speed under low SST and the wind speed bias increases with the increase in SST, making the HY-2A wind speed overestimated. The blue dashed line represents the bias between the TNNW wind speed and the ECMWF wind speed, which is always close to the 0-value line and does not appear to vary with SST. Occasional large biases also occur under high wind speed or high SST conditions, presumably due to the small amount of data used for the test and they are therefore prone to large biases.

**Figure 12:**
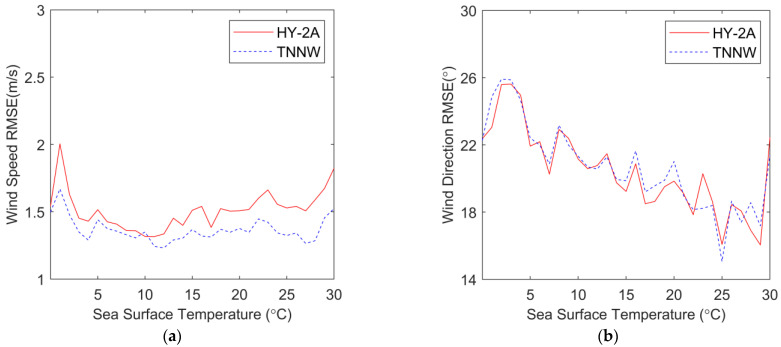
RMSEs of wind speed and wind direction as functions of ECMWF SST. (**a**) is the wind speed RMSE, (**b**) is the wind direction RMSE. Red solid lines represent HY-2A wind and blue dashed lines represent NNW wind.

Figure 12 shows the wind RMSE curves of HY-2A and NNW with ECMWF as a function of SST, Figure 12a shows the wind speed RMSE, and Figure 12b shows the wind direction RMSE. The HY-2A wind speed RMSE is between 1.34 m/s and 2.01 m/s, while the TNNW wind speed RMSE is slightly lower than that of HY-2A and the difference between them is small. The red and blue wind direction RMSE curves in Figure 12b are very close but the RMSE increases sharply at 3 °C, presumably because of the increased deviation due to fewer data used for statistical analysis. In general, the difference between the wind fields from the TNNW-corrected backscatter coefficient inversion and the ECMWF wind fields is small, indicating that TNNW has a certain degree of correction effect on the SST influence.

## 4. Discussion

The backscatter coefficients observed using the scatterometer have components of both sea surface wind and non-wind factors, and the accuracy of scatterometer wind measurements can be improved by removing the influence of secondary factors, such as SST, in the backscatter coefficients. The relationship between the sea surface wind and other factors on the backscatter coefficient is highly nonlinear, so it is very complicated and difficult to distinguish them using conventional analytical methods. However, the neural network has a very powerful learning ability and is good at generalizing the patterns existing among a large amount of multidimensional data, which is suitable for extracting the features of SST influence from the backscatter coefficients. However, neural networks are not perfect methods.

In this study, we noticed that the effect of neural network fitting depends largely on the spatial distribution of the input data and, if there are too few data with some features, it is often difficult to extract the features of that part of the data during the training of the neural network, which eventually leads to the accuracy of the neural network tending to those inputs with larger amounts of data. At the same time, neural networks need sufficient data for training and learning, otherwise they are prone to overfitting or underfitting and so on. How to balance the relationship between data volume and data distribution becomes a problem to be considered. Another limitation of neural networks is their inability to explain the predicted results. Although neural networks can provide relatively accurate predictions by learning from a large amount of data, they cannot provide a clear inference and explanation process. Although we corrected the effect of SST on the backscatter coefficient by neural network, the mechanism between the SST and scatterometer wind measurement perturbation could not be explained by the neural network.

Numerous qualitative studies have been conducted on the impact of SST on the scatterometer and it is basically clear that SST affects the sea surface roughness and the sea surface backscatter coefficient by influencing the ratio of air to seawater density, seawater viscosity, seawater dielectric constant (DC), and other factors [44,45,46]. First, the change in SST causes the change in the seawater dielectric property and the temperature and the DC of seawater are negatively correlated, while the changes in DC and backscatter coefficient are positively correlated, so there is a coupling relationship between the sea surface DC and backscatter coefficient. Second, the SST variation changes the dynamical properties of the atmosphere and seawater, which dominate the generation of surface roughness. Under the condition of fixed wind speed, there is a direct relationship between sea surface centimeter wave and seawater viscosity. The higher the viscosity of seawater, the smaller the wave height of the sea surface centimeter wave and the smaller the backscatter coefficient, while, as the SST increases, the viscosity of seawater decreases, the sea surface becomes more undulating, and the backscatter coefficient increases. The density ratio between air and seawater also decreases with increasing SST, so the drag coefficient of the wind on the sea surface is reduced, causing the backscatter coefficient to decrease.

Combining the characteristics of the variation in backscatter coefficient with SST found in our study and the analysis of the SST influence mechanism above, we infer that the changes in seawater viscosity and seawater/air density ratio dominate the SST impact on the backscatter coefficient, but since both behave in such a way that the backscatter coefficient is proportional to the change in SST, it is difficult to discern which of the two plays a larger role using simple numerical analysis. If seawater viscosity and seawater-to-air density ratio data—or either of them—are available, a more complete model of the SST-influenced backscatter coefficient can be analyzed and constructed. The limitations of the experimental conditions make it difficult to measure seawater viscosity and seawater/air density ratio directly. We hope to calculate these parameters with some indirect data in the future to help us better analyze and remove the effect of SST on the backscatter coefficient. Exploring the mechanism of the interaction between SST and sea surface wind field and constructing a physical model are directions worthy of in-depth study, but the implementation of this method is very difficult because the knowledge of the relationship between the sea surface backscattering mechanism and sea surface parameters is not complete. Therefore, there is still much room for exploration of how to resolve the effect of sea surface temperature.

Although there have been some mechanistic discussions on the effects of SST, it is still difficult to form theoretical models that can be used for scatterometer inversion of wind fields. We propose a direct correction of the backscatter coefficients using a neural network model, and different types of scatterometers can obtain a model that improves the influence of SST using neural network training. However, since it is more difficult to obtain data in the high wind speed segment, TNNW is only applicable to wind speed conditions of 4~15 m/s. Adding more scatterometer data can help TNNW to achieve a better performance. Currently, the latest HY-series Ku-band scatterometers HY-2B and HY-2C scatterometers are in operation, but we could not obtain these latest scatterometer data due to limited data access sources. The SST impact neural network model constructed using HY-2A is a preliminary exploration and, after the latest HY-2B and HY-2C data are obtained, they will be added to our model with a view to making TNNW more widely applicable.

## 5. Conclusions

In this study, we used HY-2A L2A, WindSat wind vector, and SST data to improve the SST influence on the HY-2A wind measurement using a neural network approach. In addition to the analytical verification of the results using WindSat data, we also used ECMWF wind field and SST data for secondary validation. The conclusions are as follows:

(1) The SST has a certain influence on the wind measurement accuracy of the HY-2A scatterometer and this influence is related to the polarization. In this study, we found that the temperature sensitivity of VV polarization reached a maximum of 1.17, which is more sensitive to the change of SST than HH polarization. In addition, the effect of SST is also related to the wind speed. When the wind speed is 4 m/s, the temperature sensitivity of the backscatter coefficient to the change of SST is 0.5, while the amount of change in the backscatter coefficient with the change in SST increases as the wind speed increases, and the temperature sensitivity of the backscatter coefficient of VV polarization even exceeds 1 when the wind speed is 14 m/s.
(2) The effect of SST on the HY-2A scatterometer is more obvious in the mean wind speed bias and the maximum value of mean wind speed bias between HY-2A and WindSat can reach 1.2 m/s, while the wind field inverted by the TNNW-corrected backscatter coefficient basically improves the wind speed bias so that the maximum value of mean wind speed bias does not exceed 0.5 m/s. At the same time, the RMSE of the retrieved wind field of the TNNW-improved backscatter coefficient is similar to that of the HY-2A wind field, indicating that no other deviations are introduced during the improvement process.
(3) A method was proposed to remove the influence of sea surface temperature on HY-2A scatterometer wind measurements using a neural network model—TNNW. Although this method is more suitable for satellite-borne scatterometers in operation than theoretical analysis methods that require the construction of complex models—and can improve the accuracy of HY-2A scatterometer wind measurements to some extent without rebuilding the GMF—the limitations of the neural network itself prevent us from analyzing more deeply how sea surface temperature affects the scatterometer wind measurements. A focus of our future work is to develop a more accurate model for improved scatterometer wind measurements based on an understanding of the mechanism by which sea surface temperature affects the backscatter coefficient and to apply this technique to the newer HY-series scatterometers.

## Figures and Tables

**Table 1:** Correlation coefficients of TNNW-corrected backscatter coefficients and WindSat-simulated backscatter coefficients.

WindSat Wind Speed	VV-Pol	HH-Pol
4 m/s	0.4240	0.6256
5 m/s	0.4809	0.5543
6 m/s	0.5370	0.7308
7 m/s	0.6253	0.8221
8 m/s	0.8477	0.9191
9 m/s	0.8993	0.8843
10 m/s	0.8383	0.8850
11 m/s	0.8165	0.9564
12 m/s	0.8264	0.9090
13 m/s	0.9332	0.9486
14 m/s	0.9017	0.9302

## Data Availability

Not applicable.
